# Combined effect of Polymyxin B and Tigecycline to overcome Heteroresistance in Carbapenem-Resistant Klebsiella pneumoniae

**DOI:** 10.1128/Spectrum.00152-21

**Published:** 2021-10-27

**Authors:** Yuan Tian, QiaoYu Zhang, LiRong Wen, JianSen Chen

**Affiliations:** a Department of Nosocomial Infection Control, Fujian Medical University Union Hospital, Fuzhou, Fujian, China; University of Pittsburgh School of Medicine

**Keywords:** carbapenem-resistant *Klebsiella pneumoniae*, polymyxin B, tigecycline, heteroresistance, time-kill assay

## Abstract

We assessed the prevalence of polymyxin B (PMB)- and tigecycline (TGC)-heteroresistant Klebsiella pneumoniae isolates and investigated the combined effect of PMB and TGC against dual-heteroresistant K. pneumoniae. Ninety-five nonduplicated carbapenem-resistant K. pneumoniae (CRKP) clinical isolates were collected from a tertiary-care teaching hospital in China. PCR was used to detect the resistant genes among the CRKP isolates. Population analysis profiling (PAP) was carried out to evaluate the existence of heteroresistance. A time-kill assay of PMB combined with TGC was conducted against heteroresistant K. pneumoniae strains. Real-time PCR was performed to determine the *pmrA*, *phoP*, and *acrB* expression levels. Among them, 74 isolates (77.9%) were susceptible to TGC, and 90 isolates (94.7%) were susceptible to PMB. In addition, of the TGC-susceptible isolates, 49 strains (66.2%) exhibited heteroresistant phenotypes. All of the PMB-susceptible isolates showed heteroresistant phenotypes. Forty-six isolates (48.4%) were heteroresistant to both TGC and PMB. All of the isolates carried the *bla*_KPC_ gene, and one strain carried both *bla*_KPC_ and *bla*_NDM_ genes. The time-kill assay revealed in four isolates that early bactericidal activity could be triggered by the combination of PMB and TGC, and there was no regrowth, even at a relatively lower concentration (0.125 mg/liter PMB with 1 mg/liter TGC). Upregulated expression of *pmrA*, *phoP*, and *acrB* indicated that heteroresistance could be related to two-component systems and the AcrAB-TolC efflux pump. The combination of PMB and TGC may be a treatment strategy for those infected with CRKP heteroresistant to PMB and/or TGC.

**IMPORTANCE** Tigecycline and colistin are two of the last treatment options remaining for carbapenem-resistant *Enterobacteriaceae*. Unfortunately, tigecycline resistance and colistin heteroresistance are also increasing rapidly. In the current study, we identified a high prevalence of heteroresistance to both PMB and TGC among clinical isolates of carbapenem-resistant K. pneumoniae (CRKP). The resistant subpopulations could survive pressure from TGC or PMB but were killed by the combination at a relatively low dose. It is proposed that the combination of PMB and TGC may be a treatment strategy for patients who are infected with CRKP heteroresistant to PMB or TGC.

## INTRODUCTION

Carbapenem-resistant Klebsiella pneumoniae (CRKP) has long been an intractable issue in clinical antimicrobial infections (1–3). Infections caused by CRKP usually affect patients with multiple comorbidities and are an independent predictor of mortality (4, 5). Notably, CRKP infection is not only limited to acute care hospitals but can also be found in long-term-care facilities (6).

The acquisition of carbapenem resistance in CRKP is usually related to the production of carbapenemase; *bla*_KPC_ is the most prevalent carbapenemase gene in K. pneumoniae worldwide (7, 8). Currently, the available therapeutic options are limited (9, 10). Polymyxin B (PMB) and tigecycline (TGC) seem to be used as the last-resort options to cure CRKP infection, but clinicians still need to consider several factors, such as nephrotoxicity, available plasma concentrations *in vivo*, and increasing resistance during the treatment (11–13). Vigilance should remain high even if the bacteria is identified as susceptible by the antibiotic susceptibility test. Several studies have reported that TGC-susceptible K. pneumoniae developed resistance during treatment (5, 8, 14, 15). Similarly, a recent study has found that the selective pressure of antibiotics causes bacteria to change from sensitive to resistant, which prevents the bacteria from being killed by colistin (13, 16). Microbial pathogens employ several survival strategies to avoid the body’s bactericidal defense system. The mechanisms of resistance to PMB are associated with the two-component systems PhoP/PhoQ and PmrA/PmrB, while those to TGC are associated with the overexpression of AcrAB-TolC and OqxAB efflux pumps. In addition, the ongoing discovery of heteroresistance to PMB and TGC is also of concern (17, 18).

Heteroresistance is defined as the presence of several subpopulations existing in a clinical isolate which show various levels of resistance compared with the respective parental population (13, 16). This unique phenotype lacks a conventional detection method and may be misclassified as susceptible by the routine antibiotic susceptibility testing method (19). Distinct from antibiotic tolerance or persistence, heteroresistant subpopulations can replicate stably under an antibiotic microenvironment but have slower growth and higher survival rates (19). Therefore, antibiotic monotherapy may contribute to the failure of certain antibiotic treatments (13, 20, 21). There is an urgent need to explore alternative antimicrobial therapies aimed at eliminating the regrowth of resistant subpopulations in heteroresistant strains.

Considering the serious consequences of bacterial resistance, clinicians tend to adopt combination therapy to maintain the stability of antimicrobial effects (22). However, few studies have explored the effects of antibiotics for curing heteroresistant bacterial infection, especially with multiple heteroresistance phenotypes. In the present study, we aimed to assess the prevalence of dual heteroresistance in CRKP and to determine the potential efficacy of a combination of PMB and TGC for targeting CRKP that is heteroresistant to these two antibiotic regimens *in vitro*.

## RESULTS

### Antimicrobial susceptibility testing and heteroresistance identification.

**FIG 1:**
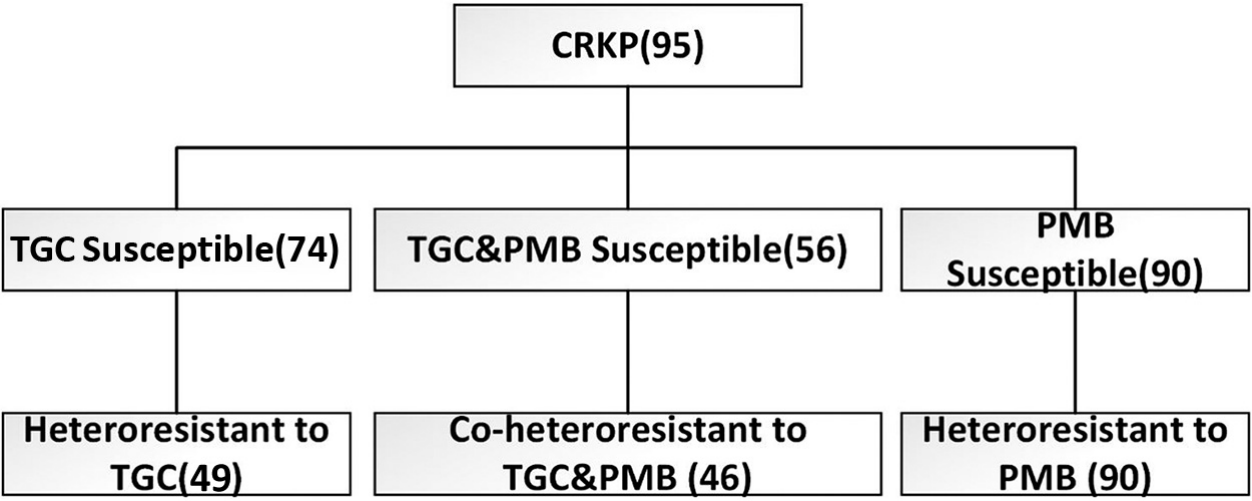
Screen for PMB and TGC heteroresistance in 95 clinical K. pneumoniae isolates.

The 95 CRKP clinical isolates we obtained for this study had resistant phenotypes to almost all antibacterial agents, except TGC and PMB. Among them, 90 (94.7%) were susceptible to PMB, and 74 (77.9%) were susceptible to TGC. It was demonstrated that 66.2% (49/74) of the TGC-susceptible isolates exhibited TGC-heteroresistant phenotypes. The more striking observation was that 100% of PMB-susceptible isolates showed PMB-heteroresistant phenotypes. Furthermore, 48.4% (46/95) of the strains were heteroresistant to both TGC and PMB (Fig. 1).

We performed PCR to identify the presence of beta-lactamases and carbapenemases. It was revealed that all the isolates carried the *bla*_KPC_ gene. Among them, 18 strains (18.9%) also carried the *bla*_CTX-M-1_ gene, and 1 strain (KP672) harbored both the *bla*_KPC_ and *bla*_NDM_ genes.

### Characteristics of four dual-heteroresistant clinical isolates.

**FIG 2:**
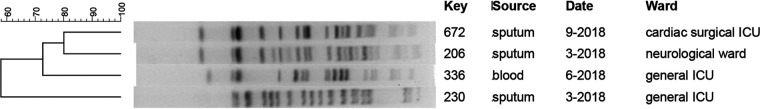
Homology analysis of 4 heteroresistant strains. The homology of the 4 heteroresistant strains was identified by PFGE. The dendrogram was developed using BioNumerics analysis software. Percent similarities are described by the unweighted pair group method using arithmetic average (UPGMA) method. ICU, intensive care unit.

**FIG 3:**
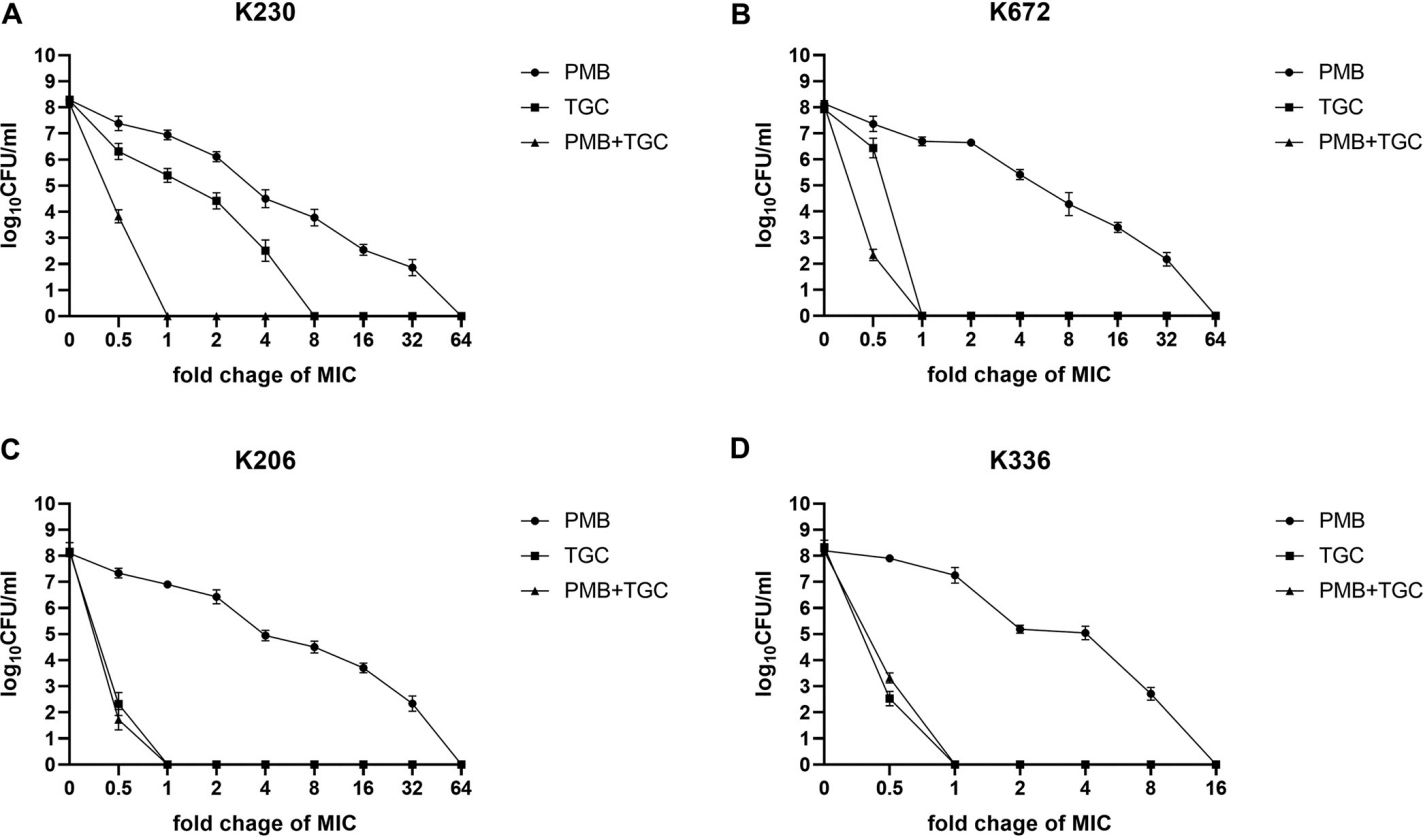
PAP confirmation of four PMB-/TGC-heteroresistant K. pneumoniae isolates. PAP is described in Materials and Methods. KP230 was heteroresistant to both PMB and TGC (A); KP672 was heteroresistant to PMB but resistant to TGC (B); and KP206 and KP336 were heteroresistant to PMB but susceptible to TGC (C, D).

Four clinical isolates with different types of resistance genes and heteroresistant phenotypes were selected for follow-up studies. These four isolates were classified into distinct patterns, indicating that they were epidemiologically unrelated (Fig. 2). All isolates displayed a multidrug-resistant (MDR) phenotype (Table 1). PAP analysis indicated that the MIC of the subpopulations grown at the highest concentration of TGC had an 8-fold (8 mg/liter) increase, and that of the subpopulations grown with PMB had a 16- to 64-fold increase (range, 16 to 32 mg/liter), compared with their respective parental strains (Fig. 3). Among the clinical isolates, KP230 was heteroresistant to both PMB and TGC, and KP672 was heteroresistant to PMB but resistant to TGC, while the other two clinical isolates (KP206 and KP336) were identified as heteroresistant only to PMB but susceptible to TGC. The frequency of PMB-resistant subpopulations in the four isolates ranged from 5.95 × 10^−7^ to 3.57 × 10^−6^, and the frequency of TGC-resistant subpopulations in KP230 was 2.20 × 10^−6^.

**TABLE 1:** Characterization of the 4 Klebsiella pneumoniae clinical isolates

		Breakpoint (MIC [mg/liter]) for:^*a*^									
Isolate	Beta-lactam resistance-associated gene(s)	PMB	TGC	IMP	CTX	FEP	CIP	AMK	GEN	Heteroresistant to PMB	Heteroresistant to TGC
KP230	*bla*_KPC_ + *bla*_CTX-M-1_	S (0.25)	S (1)	R (≥16）	R (4)	R (≥64）	R (≥4）	S (16)	I (8)	+	+
KP672	*bla*_KPC_ + *bla*_NDM_	S (0.5)	R (≥8）	R (≥16）	R (8)	R (≥64）	R (≥4）	R (≥64）	R (≥16）	+	−
KP206	*bla*_KPC_	S (0.5)	S (1)	R (≥16）	R (16)	R (≥64）	R (≥4）	R (≥64）	R (≥16）	+	−
KP336	*bla*_KPC_ + *bla*_CTX-M-1_	S (1)	S (1)	R (≥16）	R (8)	R (≥64）	R (≥4）	R (≥64）	R (≥16）	+	−

a Breakpoints and MICs calculated using the BMD method. IMP, imipenem; CTX, cefotaxime; FEP, cefepime; CIP, ciprofloxacin; AMK, amikacin; GEN, gentamicin; S, susceptible; R, resistant; I, intermediate.

### Resistant subpopulations can survive antibiotic pressure from TGC or PMB but are killed by the combination.

**FIG 4:**
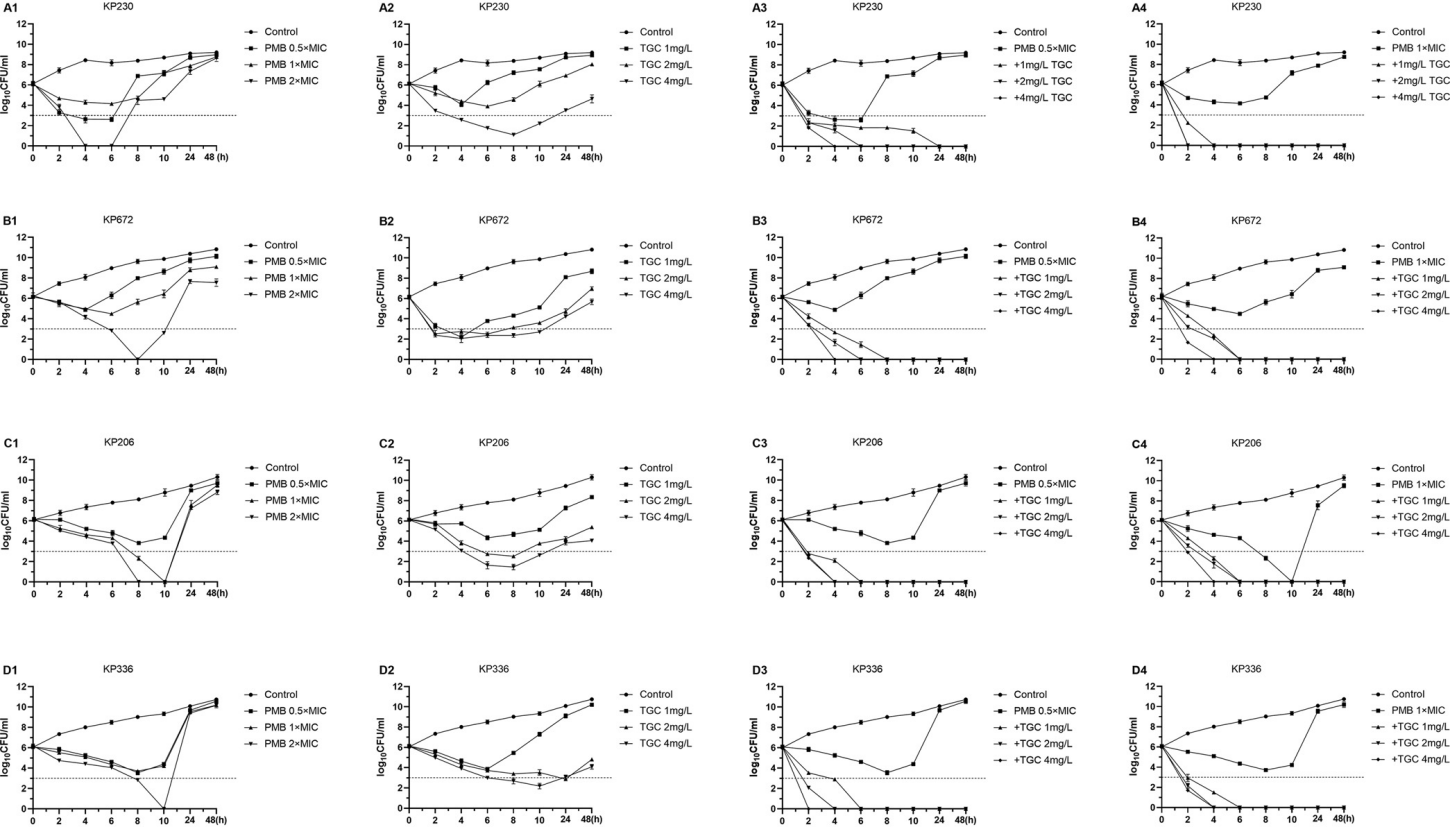
Time-kill assay of PMB or TGC monotherapy and combination treatment against K. pneumoniae clinical isolates. Four PMB- and/or TGC-heteroresistant K. pneumoniae clinical isolates were treated with different concentrations of PMB (A1 to D1), TGC (A2 to D2), or a combination of PMB and TGC with various achievable *in vivo* concentrations (A3 to D3, A4 to D4) in the time-kill analysis.

**FIG 5:**
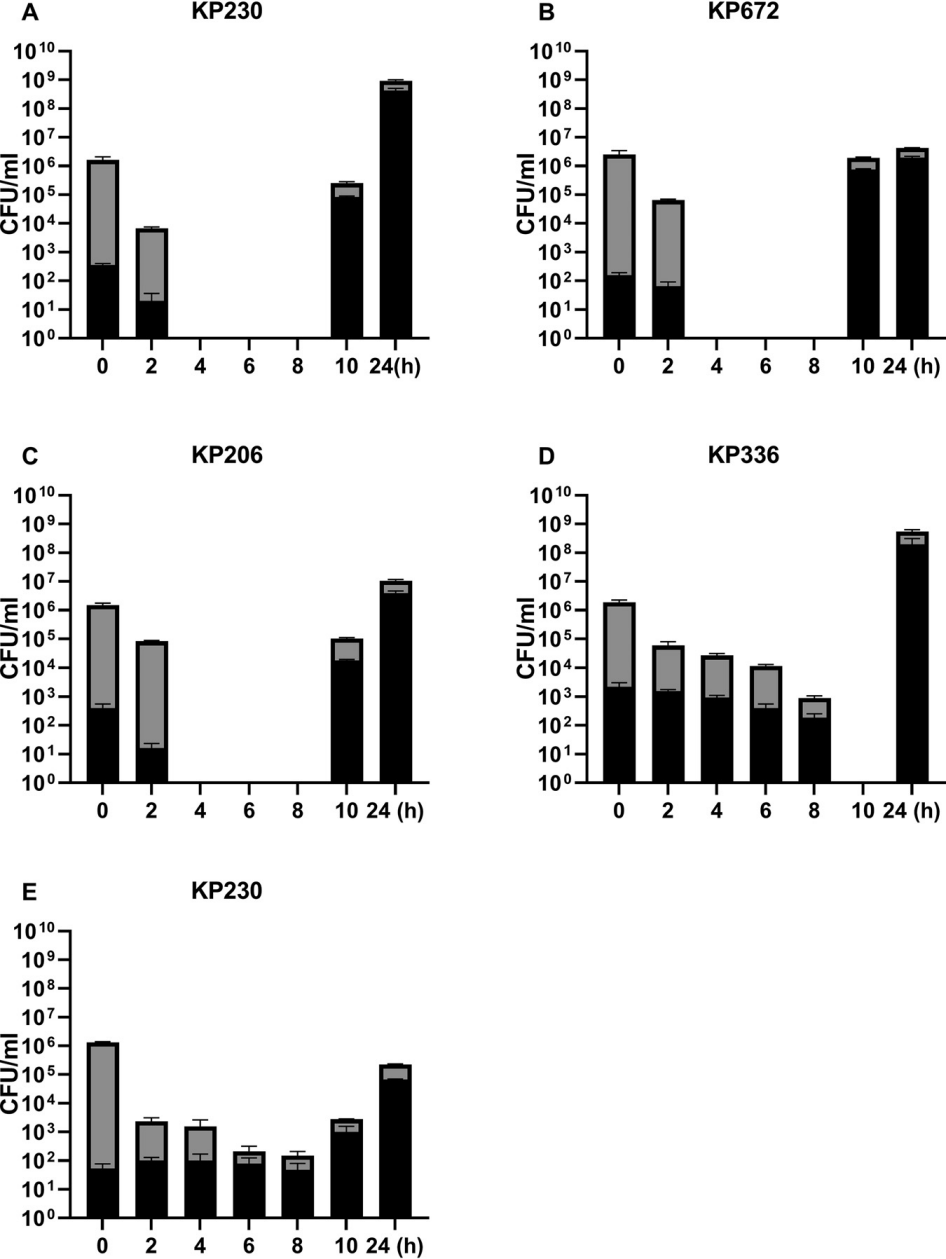
Proportion of PMB- or TGC-resistant subpopulations during the time-kill assay. The cultures were treated with 4 mg/liter PMB (A to D) or 4 mg/liter TGC (E) for 24 h. Bacteria were plated at the indicated time points to count the total CFUs (gray column) and the resistant subpopulation CFUs (black column). The error bars indicate the standard deviations of the triple-repeated experiments.

A time-kill assay was performed in 2 clinical isolates, a dual-heteroresistant strain (KP230) and a PMB-heteroresistant but TGC-resistant strain (KP672). As shown in Fig. 4, the growth of the bacteria was inhibited in the first 4 h, followed by rapid regrowth after 10 h. Treatment with a higher concentration of TGC or PMB alone killed a large proportion of susceptible subpopulations but failed to inhibit the regrowth of resistant subpopulations (Fig. 5). In addition, a time-kill assay was conducted in another two strains (KP206 and KP336) that were susceptible to TGC but heteroresistant to PMB. It demonstrated that PMB or TGC monotherapy could not achieve a bactericidal effect during prolonged treatment. Although transitorily sustained activity was observed with higher concentrations of PMB or TGC, the bacteria regrew rapidly after 10 h. However, dual treatment with PMB and TGC for all isolates may lead to an early bactericidal effect, even at lower concentrations (Fig. 4).

Next, we assessed whether the resistant subpopulations could be specifically enriched and regrow in the presence of TGC or PMB. As shown in Fig. 5, under the pressure of 4 mg/liter PMB or TGC, the most susceptible cells were eliminated rapidly in the first 2 to 4 h, followed by the resistant subpopulations rapidly increasing. The resistant subpopulations accounted for the majority of the total CFUs until 24 h. Pulsed-field gel electrophoresis (PFGE) showed high homology between native strains and their corresponding resistant subpopulations (data not shown).

### Higher expression of the genes *pmrA*, *phoP*, and *acrB* in clinical K. pneumoniae isolates heteroresistant to PMB/TGC.

**FIG 6:**
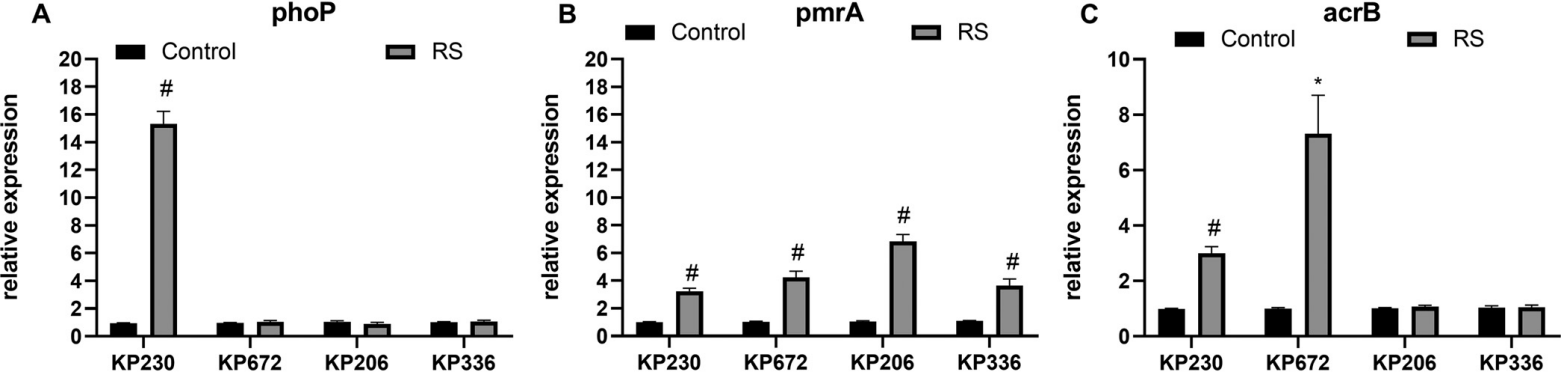
Relative expression of the genes *phoP* (A), *pmrA* (B), and *acrB* (C). The relative expression of the *phoP*, *pmrA*, and *acrB* genes between the control and their homologous RS was measured by real-time PCR and normalized to that of the *rpoB* housekeeping gene using the 2^−ΔΔCT^ method. The experiment was repeated in triplicate. Mean relative expression levels (delta *C_T_* values) and SDs are shown. A statistically significant difference between the control and RS was compared using the unpaired *t* test and represented by * (*P* < 0.01) or # (*P* < 0.001). RS, resistant subpopulations; SD, standard deviation.

To figure out the underlying mechanism related to PMB heteroresistance, we performed real-time PCR to analyze the expression of PMB resistance-associated genes. As shown in Fig. 6, compared with their parental strains, the expression of *phoP* increased 15.3-fold between the KP230 parental strain and the resistant subpopulation (Fig. 6A). In contrast, the relative expression of *pmrA* was upregulated (3.2- to 6.8-fold) in all 4 resistant subpopulations (Fig. 6B).

Next, we carried out efflux pump inhibitory assays and detected the expression of the gene *acrB*. Compared with their respective parental isolates, the MICs of KP230 and KP672 were decreased significantly, indicating the role of the AcrAB-TolC efflux pump in mediating TGC heteroresistance (Table 2). In addition, it was observed that the expression of *acrB* increased 3.0- to 7.3-fold in the TGC-resistant subpopulations of KP230 and KP672 (Fig. 6C).

**TABLE 2:** Efflux pump inhibitory assay in 4 clinical isolates

Isolate	Parental strain MIC			Resistant subpopulation MIC		
	TGC (mg/liter)	TGC + CCCP^*a*^ (mg/liter)	Fold change	TGC (mg/liter)	TGC + CCCP (mg/liter)	Fold change
KP230	1	0.25	4	8	0.125	64
KP672	8	0.5	16	NA^*b*^	NA	NA
KP206	1	1	1	NA	NA	NA
KP336	1	1	1	NA	NA	NA

a CCCP, carbonyl cyanide m-chlorophenylhydrazone.

b NA, not applicable.

## DISCUSSION

Previous studies have suggested that bacterial heteroresistance is one of the reasons for the failure of clinical antibiotic treatment (13, 23, 24). Although PMB and TGC are regarded as last-resort antibiotics to cure CRKP, it has been reported that resistance or heteroresistance to them can lead to treatment failure (25–27). Our study revealed that nearly two-thirds of TGC-susceptible isolates and all PMB-susceptible clinical CRKP isolates exhibited heteroresistant phenotypes, which was much higher than other similar investigations (26, 28). These findings suggest that heteroresistance to TGC and PMB is common in CRKP, either as single or dual heteroresistance. Therefore, there is an urgent need for a new therapeutic strategy that can inhibit the expansion of TGC- and PMB-resistant subpopulations.

In this study, we evaluated the *in vitro* effect of TGC and PMB monotherapy and combination therapy against heteroresistant CRKP. For the strains heteroresistant to PMB, killing effects were achieved during the first 4 h to 10 h of PMB monotherapy at 2× MIC, but they rapidly regrew after 10 h. This phenomenon has also been reported in previous studies (26, 29). TGC monotherapy had a transient inhibitory effect on the strains, followed by a moderate increase in growth. In short, antibiotic monotherapy was able to reduce most susceptible populations but failed to eliminate the resistant subpopulations. Many studies have shown that polytherapy has potential advantages in dealing with carbapenem-resistant isolates (7, 30, 31). Our study proved that a low dose (0.5× MIC PMB with 1 mg/liter TGC) of the antibiotic combination led to a significant decrease in the bacterial CFUs, both in single- and dual-heteroresistant strains, suggesting that combination therapy may prevent the emergence of resistant subpopulations and reduce the necessary dose of antibiotics.

In the PMB-resistant subpopulations, the MIC of PMB remained resistant but susceptible to TGC. Likewise, in the TGC-resistant subpopulations, the MIC of TGC remained resistant but susceptible to PMB. This phenomenon indicates that there are independent mechanisms responsible for TGC and PMB heteroresistance in CRKP. The molecular mechanism of PMB heteroresistance in CRKP usually involves two-component systems, such as PmrA/PmrB and PhoP/PhoQ, which is consistent with other studies (25, 26, 32). Meanwhile, TGC resistance is mainly related to the overexpression of the AcrAB-TolC efflux pump, which is supported by other research (33, 34). When resistant subpopulations were serially subcultured in liquid medium without antibiotics, the bacterial resistance phenotype remained stable. This suggests that heteroresistance is an intermediate stage of bacterial resistance and is one of the main reasons for antibiotic treatment failure.

There are several limitations in the present study. First, the combined effect of PMB and TGC was deduced from time-killing assays *in vitro*, which may not reflect accurately the combination effect *in vivo*. Second, our study only explains the independent mechanisms of PMB and TGC resistance in CRKP, and the exact mechanism of heteroresistance to PMB and TGC in CRKP needs to be further explored.

## MATERIALS AND METHODS

### Bacterial strains and characterization.

Ninety-five nonduplicate carbapenem-resistant K. pneumoniae clinical isolates were obtained from Fujian Medical University Union Hospital from January 2018 to December 2018. Bacterial identification was performed using the Vitek 2 system (bioMérieux, Marcy l’Etoile, France). The majority of the strains (55/95) were isolated from sputum samples, followed by blood (14/95) and urine (8/95), and the rest of isolates were from other sources (18/95). The isolates were stored at −80°C. Before each experiment, the isolates were resuscitated and cultured on fresh blood agar plates. The broth microdilution (BMD) method was used to determine the MIC of antibiotics (35). The susceptibility results for PMB and TGC were interpreted according to the EUCAST guidelines (PMB susceptible, ≤2 mg/liter; PMB resistant, >2 mg/liter; TGC susceptible, ≤1 mg/liter; TGC resistant, >2 mg/liter) (36). K. pneumoniae ATCC 13883 was used as the control strain.

### Population analysis profiling.

PAP was used to detect the heteroresistance of PMB and TGC (37). Briefly, 50 μl of bacterial culture was suspended in 5 ml of Luria-Bertani broth medium and incubated overnight (∼10^8^ CFU/ml). Tenfold serially diluted bacterial suspensions from 10^−8^ to 10^0^ were prepared and plated onto Mueller-Hinton (MH) agar (Oxoid, Basingstoke, UK) containing a 2-fold gradient of various concentrations of PMB or TGC from 0.25 to 64 mg/liter, respectively. Following 48 h of incubation at 37°C, the subpopulations that grew on the plates were enumerated. Heteroresistance was defined as the presence of a subpopulation of cells capable of growing at a concentration of antibiotics at least 2-fold higher than that of the antibiotic-susceptible parental strains. The frequency of heteroresistance was measured by dividing the number of colonies inoculated onto the plate with the highest concentration of antibiotic by the number of clones inoculated onto the antibiotic-free plate from the same inoculum. The limit of detection for resistant subpopulations was 20 CFU/ml. The experiments were repeated three times. To measure the stability of the resistant subpopulation, a single colony from the plate with the highest antibiotic concentration was selected and serially passaged on culture medium without antibiotics for 7 days, then reanalyzed to determine the PMB and TGC MICs (32).

### Efflux inhibition assays and detection of antibiotic-resistant genes.

The efflux pump inhibitor carbonyl cyanide m-chlorophenylhydrazone (CCCP) (10 mg/liter) (Sigma, St. Louis, USA) was applied to evaluate the effect of efflux pumps, as previously reported (38). Compared with the original bacteria, a reduction of more than 4-fold in the MIC indicates a significant inhibitory effect (39). The TIANamp bacteria DNA kit (Tiangen, Beijing, China) was used to extract bacterial whole-cell DNA according to the manufacturer’s instructions. The primers for carbapenemase (*bla*_KPC_, _-NDM_, _-VIM_, _-DIM_, _-SIM_, _-SPM_, _-GIM_, _-OXA-48_, _-AIM_, _-IMP_, and _-BIC_), extended-spectrum beta-lactamase (ESBL) (*bla*_CTX-M-1_, _-TEM_, _-SHV_, _-GES_, _-VEB_, and _-DHA_), and AmpC-like_(CMY)_ genes were adopted from previous studies (40, 41).

### Homology analysis.

Pulsed field gel electrophoresis (PFGE) was used for homology analysis of the bacterial genomes. The XbaI-digested genomic DNA (gDNA) of K. pneumoniae isolates was analyzed on the CHEF Mapper system (Bio-Rad, Hercules, CA), and the band pattern was evaluated using previously published criteria (42).

### Determination of the proportion of resistant subpopulations.

For each isolate, an initial inoculum of ∼10^6^ CFU/ml was suspended in cation-adjusted Mueller-Hinton broth (CAMHB) with 4 mg/liter PMB or TGC. The cultures were incubated at 37°C with shaking at 200 rpm. Then, 50-μl aliquots were removed, serially diluted in phosphate-buffered saline (PBS), and plated onto MH agar plates without antibiotics at various time points (0, 2, 4, 6, 8, 10, 24, and 48 h after inoculation) to determine the total CFUs. Serially diluted aliquots were also plated onto CAMHB with 4 mg/liter PMB or TGC to select the resistant subpopulations. The detection limit for resistant subpopulations was 20 CFU/ml. All experiments were performed in triplicate.

### Quantitative real-time PCR.

The PMB- and/or TGC-heteroresistant subpopulations grown on the highest-concentration drug plates were collected. A bacteria Total RNA isolation kit (Sangon, Shanghai, China) was used to extract the bacterial total RNA. A FastKing gDNA Dispelling RT SuperMix kit (Tiangen) was applied to synthesize cDNA. Real-time PCR was performed using a SuperReal PreMix Plus (SYBR green) kit (Tiangen) to evaluate the expression of *phoP*, *pmrA*, and *acrB*.

### Time-kill assay.

Four clinical isolates were selected for the time-kill assay. Briefly, bacteria mono-treated with TGC concentrations of 0.5×, 1×, or 2× MIC or PMB concentrations of 0.5×, 1×, or 2× MIC were assessed, and bacteria treated with a concentration matrix of PMB (0.5× and 1× MIC) and TGC (1, 2, and 4 mg/liter) were also evaluated. All of the concentrations used in this study can be clinically achieved *in vivo*. Bacteria were collected at different time points (0, 2, 4, 6, 8, 10, 24, and 48 h) and inoculated onto LB plates. A bactericidal effect is defined as a reduction of at least 3 log_10_ CFU/ml of bacteria compared with the original inoculum. Likewise, a synergistic effect is described as a combination of PMB and TGC, resulting in a decrease of 2 log_10_ CFU/ml of bacteria. Each experiment was performed three times.
